# Investigation of the phytochemical profiling and antioxidant, anti-diabetic, anti-inflammatory, and MDA-MB-231 cell line antiproliferative potentials of extracts from *Ephedra alata Decne*

**DOI:** 10.1038/s41598-024-65561-9

**Published:** 2024-08-06

**Authors:** Ahmad M. Al Jaafreh

**Affiliations:** Mutah University, Mutah, Jordan

**Keywords:** Antioxidant, Anti-diabetic, Anti-inflammatory, *Ephedra alata Decne*, Biochemistry, Cancer, Chemical biology, Immunology

## Abstract

Ephedra is one of the many medicinal herbs that have been used as folk/traditional medicine in Jordan and other countries to cure various illnesses. Plants of this genus are well known for their antioxidant and antibacterial properties. In this study, three different solvents were used to obtain Ephedra extracts. When evaluated, the *Ephedra alata Decne* ethanolic extract reportedly had the greatest levels of total phenolic compounds (TPC) and total flavonoid compounds (TFC). The aqueous extracts displayed the highest antioxidant activity in the DPPH and ABTS assays, demonstrating their considerable capacity to neutralize free radicals. However, when evaluated using the FRAP method, the acetone extracts showed the strongest antioxidant activity, indicating their high reducing power. LC–MS/MS, a potent method of analysis that combines the liquid chromatographic separation properties with mass spectrometry detection and identification capabilities, was used in this study to detect and measure phytochemical content of a total of 24 phenolic compounds and 16 terpene compounds present in the extracts of *Ephedra alata Decne*. Various concentrations of these chemicals were found in these extracts. The extracts’ inhibitory effects on albumin denaturation and alpha-amylase activity were also assessed; the findings demonstrated the potentials of these extracts as anti-inflammatory and anti-diabetic medicines, with the acetone extract having the lowest IC50 values in the concomitant tests (306.45 µg/ml and 851.23 µg/ml, respectively). Furthermore, the lowest IC50 value (of 364.59 ± 0.45 µg/ml) for the 80% ethanol extract demonstrated that it has the strongest antiproliferative impact regarding the MDA-MB-231 breast cancer cell line. This finding indicates that this particular extract can be potentially used to treat cancer.

## Introduction

The use of natural phytochemicals, obtained from herbal plants, in a variety of industries, including cosmetics, food, and medicine, has gained scholarly interest in the recent years. The motivation behind such interest is the desire to substitute synthetic compounds, that might be harmful or have unfavorable side effects, with natural phytochemicals. These phytochemicals are potentially safer substitutes that can be applied in several situations and entail utilizing the relevant plants’ advantageous traits. This trend is in line with the rising demand for environmentally friendly and sustainable solutions across all industries in the world.

In fact, people across the globe have traditionally relied on natural medicines, including those made from medicinal plants, especially in the less-developed countries. Throughout history, those belonging to ancient cultures have utilized local fauna and plants for their therapeutic qualities. Even though some preparations may have led to unanticipated side effects or may have operated through placebo effects, traditional therapeutic procedures have putatively included a wide variety of plant-based treatments.

In this context, the present study focuses on *Ephedra alata Decne*., sometimes locally referred to as Alanda in Arabic or Nued, which belongs to the Ephedra genus that is a member of the Ephedraceae family. The Ephedra genus contains about 67 species that are mostly found in desert areas of North Africa, Asia, Europe, and America^1^. According to one study^2^, these species are typically shrubs that have evolved in such a way that they can survive in semiarid and desert environments. A perennial plant reaching a height of more than one meter, *Ephedra alata* stands out for its intense pine-like flavor and astringent aroma. It is regarded as the angiosperms’ closest extant relative and is a member of the Gnetales plant clade. Notably, four different *Ephedra l.* species have been discovered in Jordan. These species include the following: *Ephedra foliate Boiss*, *Ephedra alata Decne*, and *Ephedra transitoria Riedl*^3,4^.

Specifically, numerous bioactive phytoconstituents are found in *Ephedra alata* (*E. alata*), including glycosides, flavonoids, phenolic compounds, and alkaloids, which reportedly contribute to the herb’s pharmacological and toxicological effects. The phytochemical makeup of *E. alata* can change depending on the particular species, the plant parts, the harvest times, and the geographic origins^5,6^. Considering their numerous therapeutic benefits and commercial importance, Ephedra species, especially *E. alata*, are highly prized in the sectors of ecology, business, and medicine. The findings of relevant studies have highlighted the significance of *E. alata* for conventional medicine and its potential with regard to further research in drug development and therapeutic drug uses^7^.

In the domain of medicine, oxidative stress caused by reactive oxygen species (ROS) is a major factor in the emergence of many chronic illnesses in human beings. According to Rechner et al.^8^, important biological molecules may be harmed by ROS, which also play a part in the etiology of illnesses such as cancer, inflammatory aging, cardiovascular disease, and diabetes. Experts claim that ROS are involved in the progression of the cell cycle, energy metabolism, survival of cells, shape of cells, adhesion between cells, cell movement, angiogenesis, as well as the maintenance and growth of tumors and metastases^9^. In this light, *E. alata* stem decoction has been historically used as a stimulant, deobstruent, and a product in the treatment of many illnesses such as gastrointestinal, kidney, bronchial, and circulatory system ailments. Moreover, it has antifungal qualities and has also been used to treat asthma episodes, in addition to being utilized in the treatment of many other diseases, including cancer.

According to the existing literature, the aqueous extracts of *E. alata* can inhibit tumor angiogenesis, induce apoptosis, prevent invasion and proliferation, and cause cell cycle arrest. Researchers in Palestine have studied the antioxidant and anti-inflammatory properties of *E. alata*^10,11^. Meanwhile, studies conducted in Tunisia^12^ have concentrated on the *E. alata* extracts’ outstanding anticancer properties with regard to the breast cancer cells MCF-7 and 4T1. Together, these studies have demonstrated that *E. alata* is great a source of bioactive substances containing anti-inflammatory, antibacterial, antioxidant, and anticancer properties. In this light, it is necessary to delve into the Jordanian Ephedra plant’s anti-inflammatory, antioxidant, and anticancer characteristics, given the scarcity of knowledge regarding its biological activity till date. Hence, this study investigated the phytochemical content and the myriad biological properties (e.g., antioxidant, anti-diabetic, anti-inflammatory, and antiproliferative potential), especially with respect to the MDA-MB-231 cell line, of the extracts gathered from *Ephedra alata Decne* grown in Jordan; moreover, it compared these data with those regarding other Ephedra species found in other regions of the world.

## Material and methods

### Plant extraction

Fresh *E. alata* aerial parts were collected from several trees located in the Al-Tafial district of Jordan at the end of March 2023. The collection was conducted in compliance with the standards and the pertinent legislation of Jordan’s institutions of governance. The voucher specimens (Mu 202307) were verified by a botanist and were stored in the Faculty of Science, Mutah University, Jordan. The aerial components were taken out and dried for 10 days in the shade. The components were pulverized using a Moulinex Miller (France) after drying. Then, 500 ml of various solvents were used to extract 50 g of the obtained powder. The various mixtures were subsequently blended for 16 h at 180 rpm at room temperature using a rotary shaker (New Brunswick Scientific, USA). Thereafter, they were exposed to sonication for 15 min at 37 °C in an ultrasonic bath (Bandelin Electronic-RK-103 H, Germany). The mixtures were then made to pass through filter paper (Whatman No. 4) and were further filtered through a syringe microfilter (0.45 m). By using a rotary evaporator (Büchi, RE 121, Switzerland), the solvents were subsequently evaporated at decreased pressure, at a temperature of 38 °C, and at 120 rpm, so that the crude extracts could be produced. After solvent evaporation, the dry solid residue that was left behind was collected and kept at a temperature of 4 °C for future examination. Notably, the yield percent was calculated as follows:

$$\text{Yield\%}=(\text{amount \, of \, dry \, extract }(\text{g}) /\text{ amount \, of \, dry \, sample \, used }(\text{g}))*100 {\%}$$

### Determination of total phenolic content (TPC)

The estimation of the total phenolic concentration in Ephedra extracts can be achieved by the Folin-Ciocalteu method, according to an existing study^13^. Following this method, in this study the crude extracts were dissolved in 15 ml of dimethyl sulfoxide (DMSO). Then, 2.5 ml of 0.2 N Folin-Ciocalteu reagent was added to 500 l of Ephedra extract. The resulting mixture was blended with 2 ml of sodium carbonate solution (7.5% w/v in deionized water) after being kept for five minutes at room temperature. A UV/Visible spectrophotometer (HITACHI U-5100 UV–VIS spectrophotometer) was used to measure its absorbance at 760 nm; the combination was incubated for 120 min at room temperature and in a dark setting. Further, gallic acid (with concentrations values of 0.01–0.05 mg/ml) was used to construct the standard calibration curve. Importantly, milligrams (mg) of gallic acid equivalent per gram of dried Ephedra extracts served as a measure of the given extract’s overall phenolic content.

### Determination of total flavonoid content (TFC)

According to a previous study, a spectrophotometric approach can be used to measure the total quantity of flavonoids found in Ephedra extracts^14^. This procedure involves mixing 0.5 ml of an Ephedra extract solution with 300 µl of a sodium nitrite solution at a concentration of 5 g/l. Following this method, 300 µl of a 1 g/l aluminum chloride solution was added to the mixture after five minutes. Six minutes later, 2 ml of 1 M sodium hydroxide was added to the mixture. The entire mixture volume was made to reach 10 ml and was sonicated with distilled water. At 510 nm, its absorbance was measured in comparison to that of a water blank using a UV/Visible spectrophotometer (HITACHI U-5100 UV–VIS spectrophotometer). A calibration curve was also made by preparing a rutin solution (0–200 µg/ml). For each gram of dried Ephedra, the amount of flavonoids in each Ephedra extract was measured and noted (in terms of milligrams of rutin equivalent).

### Determination of antioxidant activity of *E. alata* extracts

#### DPPH radical scavenging activity

In a 2022 study^15^, a novel approach was used to assess the scavenging efficacy of Ephedra extracts. Here, 100 µl of various amounts of Ephedra extracts were dissolved in 1.9 ml of methanol solution of DPPH. After giving the mixture a robust shake, the mixture was kept standing for 30 min at room temperature (25 °C). A UV/Visible spectrophotometer (HITACHI U-5100 UV–VIS spectrophotometer) was subsequently used to measure the mixture’s absorbance at 517 nm in comparison to a blank (the latter was generated using a methanolic DPPH dilution). Notably, positive controls were used, e.g., gallic acid. In turn, the following formula used to determine the DPPH radical scavenging activity:

$$\text{DPPH \, radical \, scavenging \, activity }\left({\%}\right)=\left[\frac{\left(\text{Ac }-\text{ As}\right)}{Ac}\right]{\times }100 ,$$

where As denotes the absorbance of the sample and Ac denotes the control absorbance.

#### ABTS free radical scavenging assay

To create a 7 mM ABTS solution, in accordance with a previous study^16^, 2.45 mM potassium persulfate was combined with ABTS stock solution and left to sit in the dark for 16 h at room temperature. The resultant ABTS solution was subsequently diluted with ethanol and measured using a UV/Visible spectrophotometer (HITACHI U-5100 UV–VIS spectrophotometer) to achieve an absorbance at 734 nm of 0.70 ± 0.02. For the ABTS radical scavenging activity experiment, 2 ml of the diluted ABTS solution was mixed with 20 µl of various trolox concentrations, Ephedra extracts, or solvent (control). Following the mixture, the absorbance reading was measured precisely six minutes later. In turn, at least three different determinations were made. The following formula was used to determine the ABTS radical scavenging activity:

$$\text{ABTS \,  radical \,  scavenging \, activity }\left({\%}\right)=\left[\frac{\left(\text{Ac }-\text{ As}\right)}{Ac}\right]{\times }100 ,$$

where As denotes the absorbance of the sample and Ac denotes the control absorbance.

#### Ferric reducing antioxidant power assay (FRAP)

In this study, the ferric reducing antioxidant power (FRAP) assay was conducted in accordance with the approach used by a relevant previous study^17^. The Ephedra plant extracts were mixed with 3 ml of the FRAP reagent, which was composed of 20 mmol/l of FeCl_3_ solution, 300 mmol/l of acetate buffer (pH 3.6), and 10 mmol/l of TPTZ solution in a ratio of 1:10:1 (v/v/v). The absorbance at 593 nm was measured by a HITACHI U-5100 UV–VIS spectrophotometer after the reaction had been allowed to continue for 10 min at room temperature. Ascorbic acid (As) was utilized as a reference substance, having a concentration range of 25–500 µg/ml. Thereafter, ascorbic acid equivalents (AsE) per gram of dried Ephedra extract were used to express the assay results.

#### Albumin denaturation inhibition assay

Following a previous study^18^, with a total volume of 5.0 ml for each sample, successive dilutions of the *E. alata* extract were achieved for the purpose of conducting the protein denaturation inhibition experiment (ranging from 50 g/ml to 1000 µg/ml). Moreover, fresh hen egg albumin and 2.8 mL of phosphate-buffered saline (pH 6.4) were used to make the reaction mixtures. Thereafter, 2 mL of *E. alata* extracts of the corresponding concentrations were gently included in each reaction mixture. Distilled water was used as the negative control. After being incubated for 15–20 min at 37 °C, the reaction mixtures were heated in a water bath at 70 °C for 5 min. Then, at room temperature, the reaction mixture was allowed to cool for 15 min. A colorimeter was used to test the reaction mixture’s absorbance at various concentrations (1000 µg/ml, 500 µg/ml, 200 µg/ml, 100 µg/ml, and 50 µg/ml) both before and after denaturation. Three repetitions of the test were made, and the mean absorbance of the mixture was noted across the these tests.

The following formula was used to determine the proportion of protein denaturation that was inhibited in comparison to the control:

$$\text{Percentage \, inhibition \, activity }\left({\%}\right)=\left[\frac{\left(\text{Ac }-\text{ As}\right)}{Ac}\right]{\times }100 ,$$

where As denotes the absorbance of the sample and Ac denotes the control absorbance.

#### The α-amylase inhibitory assay

A modified version of the industry-standard methodology was applied in the α-amylase inhibitory assay, in accordance with a previous study^19^. The substrate solution was made by dissolving 2 mg of starch azure in 200 µl of 0.5 M Tris–HCl buffer (pH 6.9) containing 10 mM of CaCl_2_. The tubes holding the substrate solution underwent a 5-min pre-incubation at 37 °C and a 5-min boil.

Thereafter, Ephedra extracts were dissolved in DMSO to create extract concentrations of 25 g/ml, 50 g/ml, 100 g/ml, 150 g/ml, 200 g/ml, and 250 g/ml. Then, 0.2 ml of concentrated plant extract was added to the tube containing 0.1 ml of porcine pancreatic amylase in Tris–HCl buffer (2 units/ml); and 10 min of the following reaction were spent conducting the same at 37 °C. By adding 0.5 ml of 50% acetic acid to each tube, the reaction was subsequently stopped. Then, the reaction mixture was centrifuged at 4 °C for 5 min at 3000 rpm. Using a spectrophotometer, the absorbance of the supernatant was determined to be at 595 nm. Importantly, the same abovementioned process was used to observe how different plant extracts in acetone and water affected the functional ability of α-amylase. Moreover, three separate runs of the experiments were completed, and the mean absorbance across the runs was noted. The level of inhibition was calculated as follows:

$$\text{Inhibition }\left({\%}\right)=100- \% \text{ reaction},$$

where % reaction (at *t* = 3 min) = (mean maltose in sample / mean maltose in control) × 100.

Further, the IC50 value, which is known as the amount of plant extract required to inhibit 50% of α-amylase activity under the studied conditions, was calculated in this study. Specifically, the plant extracts’ inhibitory effects on α-amylase and their IC50 values were calculated.

#### Antiproliferation activity of *Ephedra*

To observe the antiproliferation acitivity of Ephedra, MDA 231 triple-negative cells were cultured in 10% fetal bovine serum (FBS) DMEM media with 1% l-glutamine and 1% penicillin–streptomycin, in an incubator with 5% CO_2_ at 37 °C.

As recommended by a previous study^20^, the MTT (3-(4,5-dimethylthiazol-2-yl)-2,5-diphenyl tetrazolium bromide) assay was applied to determine cell viability. A colorimetric assay called MTT was used to count the number of active cells, which was used to quantify cellular growth, survival, and proliferation. For the same purpose, 200 mg/ml of Ephedra extracts were produced as a stock solution in DMSO and kept at 20 °C. To keep the final DMSO concentration below 0.1%, the stock solution was diluted with the DMEM medium before the extracts were used in the cytotoxicity assay.

Thereafter, in 96-well plates, 10,000 cells were seeded with 100 l of medium for the MTT assay, being subsequently incubated at 37 °C for 24 h. Then, the cells were exposed to various Ephedra extract doses for 24 h. After the treatment, each well received 10 l of an MTT solution in PBS (5 mg/ml), which was then incubated for 4 h at 37 °C. After carefully removing the supernatant, 100 l of DMSO was applied to each well. On a microplate shaker, all the plates were subsequently shaken for 5 min. Thermo-Fisher-Scientific (Waltham, Massachusetts, USA) provided a Microplate Reader that was used to detect the absorbance at 595 nm, aimed at calculating cell viability. Using the EXCEL application, the mean of triplicate experiments for each treatment was calculated in order to measure the extract concentration necessary for 50% suppression of cell viability (IC50).

#### LC–MS\MS for determination of chemical compositions

Using a liquid chromatographic system (LC-8030, Shimadzu, Japan), bioactive components in the extracted samples were qualitatively analyzed in this study. The column temperature control (CTO-30A), Degasser (DGU-5A), auto-sampler with cooler (S11-30 AC), quaternary solvent delivery pump (LC-30 AD), and system controller (CBM-20A) were all included as components of this system. For the purpose of separation, an Agilent Zorbax Eclipse XDB-C18 column (with a length of 150 mm, an internal diameter of 2.1 mm, and a film thickness of 3.5 m) was used. Formic acid solutions A and B, each containing 0.1% aqueous formic acid and 0.1% acetonitrile, constituted the mobile phase. Under gradient circumstances, the elution was carried out by first holding solvent A at 5% for 5 min, then increasing solvent B linearly from 5 to 100% over 15 min, and thereafter holding solvent B at 100% for 5 min. In this regard, a flow rate of 0.5 ml/min was used. Notably, the column was cleaned and re-calibrated for 15 min following each cycle. A column with a 35 °C temperature and an injection volume of 50 L was used as an additional elution condition. To monitor the eluent, a positive ion mode Shimadzu LC–MS 8030 electrospray ionization mass spectrometer (ESI–MS) was used. This mass spectrometer scanned the mass-to-charge ratio (m/z) from 100 to 1000. Meanwhile, SKIMMER ran at 65 V and ESI ran at 125 V. During the drying procedure, (99.999%) high-purity nitrogen (as the drying gas) was used in a fragment nebulizer operating at 45 psi and at 350 °C. Notably, the analysis employed a 0.1% formic acid solution as a blank.

### Statistical analysis

In this study, the data analysis was performed using Microsoft Excel (Windows 10). Significant statistical differences were evaluated using the one-way analysis of variance (ANOVA).

## Results and discussion

Phenolic molecules are essential secondary metabolites in plant systems that support plant growth, resilience to stress, antioxidant action, and pigment formation. Flavonoids are phenolic chemicals that are crucial to preventing oxidative damage to macromolecules in biological systems. The principal active phytocompounds can be separated and concentrated based on their polarity by using various solvents to extract these plant components. For instance, extraction using 80% ethanol typically results in extracts with medium-level polarity that are rich in active ingredients. This implies that the biological action of these extracts may owe to their phytochemical makeup. In fact, researchers can learn more about the diverse phytochemical components of different extracts and their potential therapeutic effects by using solvent extraction techniques.

Here, one must remember that the chemical makeup and biological traits of the relevant extracted substances can be strongly impacted by the choice of extraction solvent. In this study, polarity solvents were utilized to extract putative bioactive components from *E. alata* using water, acetone, and 80% ethanol. According to the findings in Table 1, acetone and ethanol gave lesser yields (2.34% and 2.84%, respectively), while the water extraction had the highest yield (3.88%). The results were in line with the findings of an earlier study^10^.

**Table 1:** Phytochemical content and yield (%) of *E. alata* extracts.

Extract	TPC (GAE mg/g)	TF (QE mg/g)	Yield (%)
Ethanol	84.44 ± 1	39.43 ± 0.42	2.34
Aqueous	83.54 ± 2.9	28.83 ± 1.81	3.88
Acetone	63.69 ± 0.95	29.42 ± 1.69	2.84

The results of total phenol content were expressed in terms of gallic acid equivalent (mg/g), while total flavonoid content was expressed as quercetin equivalent (mg/g of extract); finally, the extract yield was expressed as a percent of the weight of extract per 100 g of dry weight plant. The results are presented in the table as averages for triplicate measurements ± standard deviations (n = 3).

Moreover, different solvents with various polarities can produce different extracts with various biological activities. It should be emphasized that due to differences in extraction procedures and environmental conditions, comparisons of extraction yields between studies can be difficult to achieve^21^. In this study, the total phenolic contents in ethanol, acetone, and water extracts of *E. alata* were calculated. The total phenolic contents of these extracts varied greatly, with the ethanol extract having the highest content (84.44 mg GAE/g of extract), the water extract having the second-highest content (83.54 mg GAE/g extract), and the acetone extract having the lowest content (63.69 mg GAE/g extract). Meanwhile, the total flavonoid content of the *E. alata* ethanol extract was found to be higher (39.430.42 mg QE/g of extract) than that of the acetone extract (29.421.69 mg QE/g of extract) as well as that of the water extract (28.831.81 mg QE/g of extract).

Here, one must bear in mind that numerous plant species naturally contain flavonoids as well as other phenolic chemicals which are renowned for their healing properties. According to several studies^22–24^, such chemicals have a wide range of advantageous features, such as antioxidant, antimicrobial, anti-inflammatory, anti-diabetic, cancer-preventive, antithrombotic, and anti-ischemic properties. They are frequently utilized as all-natural preservatives in common meals, medications, and cosmetics^25^.

In this study, the DPPH radical scavenging experiment was carried out to assess the antioxidant capacities of the various *E. alata* extracts (Table 2). A strong radical scavenging action was shown by the aqueous extract, with 90% inhibition being observed (119 µg/g GAE). Specifically, the acetone extract had a scavenging activity of 76.71% (100.1 µg/g GAE), while the ethanol extract had a scavenging activity of 70.28% (90 µg/g GAE).

**Table 2:** Antioxidant activity of *E. alata* extracts.

Extract	DPPH		ABTS		FRAP
	% inhibition	GAE (µg/g)	% inhibition	TE (µg/g)	AsE (µg/g)
Ethanol	70.28 ± 0.74	119	75.85 ± 1.55	70.13	40.75 ± 4.7
Aqueous	90.19 ± 0.58	90	94.17 ± 0.58	75.25	24.86 ± 1.04
Acetone	76.71 ± 0.53	100.1	81.2 ± 1.08	89.3	42.61 ± 2.46

In this study, the DPPH scavenging activity was expressed in terms of gallic acid equivalent (µg/g extract), the result of ferric ion reducing power was expressed as ascorbic acid equivalent (µg/g extract), and the result of ABTS scavenging was expressed as trolox equivalent (µg/g extract). The results in the table are shown as averages for triplicate measurements ± standard deviations (n = 3).

Notably, both the ABTS free radical scavenging test and the DPPH scavenging capabilities test had a similar pattern. A high 94% suppression (89.3 µg/g TE) of the radical scavenging activity was seen in the aqueous extract. As per the DPPH radical scavenging experiment in this study, the acetone extract had a scavenging activity of 81.19% (75.25 µg/g TE), in contrast to the ethanol extract’s 75.85% (70.13 µg/g TE) scavenging activity. In line with findings of the ABTS experiment, DPPH also demonstrated that the antioxidant capacity of Ephedra plant extracts varied according to the solvent utilized^26^.

In a previous study^10^ using Palestinian *E. alata*, higher polyphenol content and better antioxidant activity were produced by utilizing more polar solvents. In that investigation, the hydroethanolic extract showed the strongest radical scavenging activity. Similarly, this investigation found that there were significant associations between polyphenols and antioxidant activity when measured by the FRAP, DPPH, and ABTS radical scavenging assays regarding both aqueous and ethanol–water extracts. A different study on *E. alata* from Jenin, Palestine, found that the methanolic extract had lower EC50 values and greater radical scavenging activity^27^. These results aligned with the higher level of antioxidant activity seen in radical scavenging activity assays. In the present study, while the aqueous extract had a lesser concentration of phenolic components than the ethanolic extract, it had a comparatively higher level of antioxidant activity, which was consistent with the conclusions made by Alali’s study^28^. Similarly, in this study more phenolic components were recovered from the aqueous extract than from the acetone extract, and the antioxidant activity was reportedly stronger in the aqueous extract.

Moreover, the acetone extract in this study showed a high reducing power as per the FRAP assay (42.61 AsE µg/g), demonstrating its capacity to contribute electrons and reduce ferric ions. The reducing powers of the ethanol and aqueous extracts, in contrast, were lower (40.75 AsE µg/g and 24.86 AsE µg/g, respectively). The results of the FRAP assay indicated that the acetone extract promised more potent antioxidant activity in terms of its ability to donate electrons, whereas the ethanol and water extracts could have relatively lower antioxidant activity in this regard. Importantly, one must remember that various antioxidant assays may produce inconsistent results, and the choice of assay can affect the pattern of antioxidant activity that is detected among the extracts.

In another study, where three Iranian wild-culture Ephedra species (*E. strobilacea Bunge* and *E. pachyclada Boiss*.) were examined, an assessment using the FRAP assay^29^ led to the discovery that the methanolic extract of *E. strobilacea* displayed higher antioxidant activity than the other two species. In this respect, it is crucial to remember that different Ephedra species and even the various tissues of *E. alata* might differ in terms of the makeup of bioactive components^30^. Their antioxidant capacity may also be affected by the presence of synergistic or inhibitory interactions between biomolecules^31–33^. Additionally, the particular extraction solvent that is used can affect the degree to which extracts are taken from the aerial parts and the stems of Ephedra species such as *E. gerardiana and E. chilensis* in order to scavenge the DPPH radical^34^. In fact, according to recent studies, the amount and chemical makeup of phenolic compounds can affect how well they perform as antioxidants when tested using the DPPH or ABTS assays. The molecular weight, hydroxyl substitutions, and aromatic rings of phenolic substances together affect how well they can scavenge free radicals. These results show that smaller phenolic compounds have more inhibitory effects upon DPPH, while larger phenolic compounds have stronger inhibitory effects upon ABTS^35,36^.

In addition, ethanol extracts with a higher concentration of phenolic substances may have additive or synergistic effects in terms of antioxidant activity^37^. Further studies are necessary to fully comprehend the connection between phenolic composition and antioxidant activity as these results imply that both the size and quantity of phenolic compounds in extracts can affect their antioxidant capacity.

In this study, *E. alata* extracts underwent LC–MS/MS analysis, which identified the presence of 65 secondary metabolites in total, including 15 flavonoids, 9 phenolic acids, 16 terpinenes, and 25 additional compounds. Tables 3 and 4 focus primarily on the phenolic substances and terpenes found in different *E. alata* preparations. Notably, the 80% ethanol crude extract contained a total of 24 phenolic compounds, including 9 phenolic acids (chlorogenic acid, quinic acid, gallic acid, syringic acid, caffeic acid, trans-cinnamic acid, and trans-ferulic acid), 3 flavones (luteolin, apigenin, and cirsiliol), 2 flavonols (quercetin and kaempferol), and 3 flavan-3-ols (rutin, catechin, and (−)-epicatechin).

**Table 3:** Phytochemical content of *E. alata* extracts, phenolic and flavonoid contents in the *E. alata* (80%) hydroethanolic, water, and acetone crude extracts (% used to measure phytochemical content).

Name	80% EOH	Water	Acetone	Formula
Gallic acid	6.2	1.4	2.2	C_7_H_6_O_5_
Chlorogenic acid	14.4	2.3	3.4	C_16_H_18_O_9_
Trans ferulic acid	0.3	0.2	0.6	C_10_H_10_O_4_
Caffeic acid	0.5	0.3	0.4	C_9_H_8_O_4_
Quinic acid	6.1	5.9	1.9	C_7_H_12_O_6_
Trans-Cinnamic acid	0.9	4.1	2.3	C_9_H_8_O_2_
p-Coumaric acid	0.1	0.2	4.5	C_9_H_8_O_3_
Coumarin	0.9	0	1.7	C_9_H_6_O_2_
Syringic acid	0.1	0	0.8	C_9_H_10_O_5_
Naringenin	0.3	0.9	0.7	C_15_H_12_O_5_
Rutin	2.3	1.1	2.6	C_27_H_30_O_16_
Protocatechuic acid	0.3	2.1	0.6	C_7_H_6_O_4_
Luteolin	0.4	0.1	0.8	C_15_H_10_O_6_
Apigenin 7-O-glucoside	0.9	0.8	1.5	C_26_H_28_O_14_
Kaempferol	0.9	0	1.3	C_15_H_10_O_6_
Cirsiliol	0.9	0.1	0	C_17_H_14_O_7_
Apigenin	0.6	0.3	0.2	C_15_H_10_O_5_
(+)-Catechin	1.4	2.9	3.1	C_15_H_14_O_6_
Apigetrin	0.1	0.2	0.9	C_21_H_20_O_10_
(−)-Epicatechin	11.8	3.3	4.3	_C15H14O6_
Quercitrin	0.9	4.9	5.6	C_21_H_20_O_11_
Naringin	1.2	7.2	1.3	C_27_H_32_O_14_
Quercetin	4.4	3.2	2.4	C_15_H_10_O_7_
Cynaroside	0.4	1.1	0.8	C_21_H_20_O_11_
Total	56.3%	42.6%	43.9%	–
Phenolic compounds	29.5%	14.4%	17.8%	–
Flavonoids	26.8%	28.2%	26.1%	–

**Table 4:** Phytochemical content of *E. alata* extracts, terpenes contents of the *E. alata* (80%) hydroethanolic, water, and acetone crude extracts (% used to measure phytochemical content).

Compound name	80% EOH	Water	Acetone	Formula
Myrcene	6.9	0	7	C_10_H_16_
Cymene-8-ol	0.5	2.6	1.7	C_10_H_14_O
α-Pinene	13.4	1.9	11.4	C_10_H_16_
Piperitone	0.9	0.7	1.9	C_10_H_16_O
Terpinen-4-ol	2.1	2.1	1.8	C_10_H_18_O
Cadalene	0.5	0.5	3.5	C_15_H_18_
1,8-Cineole	6.4	0.7	3.1	C_10_H_18_O
Chrysanthenone	2.8	0.7	0	C_10_H_15_O
α-Terpineol	0.3	1	0.7	C_10_H_18_O
Phellandral	0.5	0.2	0.7	C_10_H_16_O
Limonene	0.3	0	4.1	C_10_H_16_
Diosphenol	0.4	0.4	0	C_10_H_16_O_2_
Phytol	0.9	0.2	6.9	C_20_H_40_
Terpinolene	0.5	0	1.5	C_10_H_16_
α-Terpinyl acetate	1.6	1.6	1.5	C_12_H_20_O_2_
p-Cymene	0.9	0.3	2.7	C_10_H_14_
Tricyclene	0.9	0.4	5	C_10_H_16_
Total	39.8%	13.3%	53.5%	

Significantly, the variety, quantity, and composition of phenolic compounds present in the extracts might have been affected by the extraction solvent that was chosen. There were 24 phenolic compounds obtained in the 80% ethanol crude extract of *E. alata*, with chlorogenic acid (14.4%) having the highest concentration among them. Out of the 24 identified phenolic compounds found in the 80% ethanolic extract, 21 were also present in the aqueous extract and 23 were present in the acetone extract. Except for cirsiliol, which was not found in the acetone extract but was found in the ethanolic extract, the chemical profiles of the ethanolic and acetone extracts were deemed similar. Chlorogenic acid (14.4%), (−)-epicatechin (11.8%), gallic acid (6.2%), and quinic acid (6.1%) were found to be the main phenolic components in the hydro-ethanol extract; significant concentrations of other substances, including (+)-catechin (1.4%), naringin (1.2%), quercetin (4.4%), and rutin (2.3%), were also found.

The predominant phenolic substances in the aqueous extract were quercetin (4.9%), trans-cinnamic acid (4.1%), quinic acid (5. 9%), and naringin (7.2%). Additionally found in considerable concentrations were (−)-epicatechin, quercetin, (+)-catechin, chlorogenic acid, protocatechuic acid, rutin, and gallic acid. There was no evidence of the presence of coumarin, syringic acid, or kaempferol in the aqueous extract. Meanwhile, quercitrin (5.6%), p-coumaric acid (4.3%), (−)-epicatechin (3.9%), chlorogenic acid (3.4%), and (+)-catechin (3.1%) were the significant phenolic components found in the acetone extract; however, there was no trace cirsiliol in this extract. There were also significant concentrations of rutin, quercetin, trans-cinnamic acid, gallic acid, quinic acid, coumarin, apigenin-7-O-glucoside, naringin, and kaempferol in this extract. The levels of the other substances ranged from 0.9 to 0.2%, which were deemed comparatively less in the terms of proportion.

Furthermore, the three main terpene components in the ethanolic extract of *E. alata* were 1,8-cineole (6.4%), myrcene (6.9%), and pinene (13.4%). Except for chrysanthenone, terpinen-4-ol, and terpinyl acetate, which were detected at percentage levels of 2.8%, 2.1%, and 1.6%, respectively, the remaining terpene compounds were all present in smaller concentrations, ranging from 0.3 to 0.9%. Moreover, cymene-8-ol (2.6%), terpinen-4-ol (2.1%), α-pinene (1.9%), α-terpinyl acetate (1.6%), and α-terpineol (1%) were the major terpene components found in the aqueous extract. However, terpinolene, myrcene, and limonene were not found in this extract. Further, the primary terpene chemicals found in the acetone extract were pinene, myrcene, phytol, tricyclene, and limonene, which were each detected to be present in concentrations of 11.4%, 7%, 6.9%, 5%, and 4.1%, respectively. While terpineol and pellandral had the lowest concentration (0.7% each), chrysanthenone and diosphenol were simply not found in this extract. The concentrations of the other terpene compounds ranged from 1.5 to 3.5%.

There is a paucity of currently accessible information about the phenolic makeup of *E. alata*. Till date, kaempferol-3-O-rhamnoside and quercetin-3-O-rhamnoside have been found in *E. alata* growing in the Egyptian desert, according to earlier studies^38^. Quercetin has also been discovered in dietary supplements, including American-made Ephedra products^39^. The primary components of the Algerian species of *E. alata* have also found to be isoflavones and flavonol derivatives, with hydroxypuerarin isomer 1 representing the predominant molecule^40^. In the current study, epicatechin and chlorogenic acid were both present in large concentrations in the ethanol and water extracts of the *E. alata* used (11.8% and 14.4% in ethanol extract, 3.3% and 2.3% in water extract, respectively). These substances most likely played a part in the antioxidant potential that was observed and quantified by this study’s ABTS assay. The water extract did not contain many additional phenolic components; however, the substantial antioxidant activity (94.17%) demonstrated the value of the ABTS method to gauge the antioxidant potential of extracts. Recent investigations have also highlighted the inhibitory potential of these bioactive compounds as antioxidants, antimicrobials, and anticarcinogens^41,42^. At the same time, researchers^43,44^ have demonstrated that powerful antioxidant-rich extracts like gallic acid and caffeic acid can scavenge free radicals. In fact, apigenin and catechin, two phenolic compounds with high molecular weights, may be the cause of the potent scavenging to the ABTS radical. These findings concur with previous research findings^22^. Additionally, in a recent study, apigenin reportedly reduced the risk of tumor development^45^. Moreover, experts have claimed that the presence of ferulic acid, p-coumaric acid, and syringic acid may contribute to the observed extract’s significant antioxidant activity against DPPH.

Despite having a higher phenolic content, the ethanolic extract’s antioxidant potential was shown to be lower than that of the water extract. These extracts may have lesser amounts of specific phenolic chemicals, which are necessary for the extracts’ overall antioxidant ability; this fact could account for the observed differential. Indeed, experts have noted that each extract’s composition can affect its antioxidant-active level. Contrary to the findings of many studies^23,24^, this result indicates that plants with higher total phenolic component concentrations have stronger antioxidant activities. However, it can be difficult to pinpoint a single element, whether it is a significant or minor constituent, that has a level of antioxidant power akin to that of a complex mixture. Understanding the entire antioxidant capacity attained requires taking into account and further analyzing each constituent’s capacity for scavenging^46^.

As per another study, drugs that can prevent the accumulation of denatured aggregates of protein and protein condensation are beneficial for conditions such as rheumatic disorders, cataracts, and Alzheimer’s disease as the utilized proteins, which are denatured, serve as inflammatory mediators^47^. This study’s findings confirm Ephedra’s traditional use for the treatment of several painful and inflammatory illnesses. One or more of the Ephedra components may be responsible for its analgesic and anti-inflammatory activities, including phenolic compounds, tannins, flavonoids, and phytosterols. In this study, all of the compounds gathered from *E. alata* demonstrated anti-inflammatory effectiveness by dramatically lowering protein denaturation in the albumin denaturation inhibition assay.

**Figure 1:**
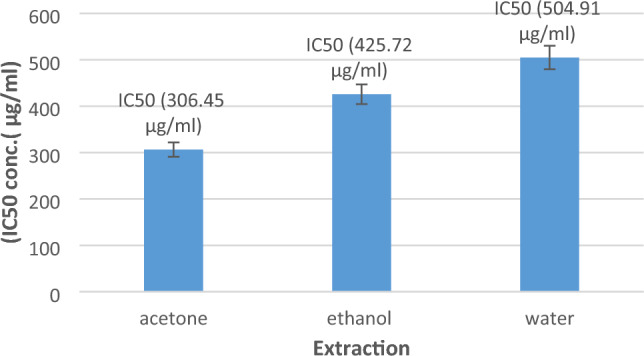
Albumin denaturation inhibition activity of *E. alata* extracts. In this figure, the results of albumin denaturation inhibition are expressed as IC50 (µg/ml); the results are shown as averages for triplicate measurements ± standard deviations (n = 3).

In fact, the albumin denaturation inhibition assay’s findings demonstrated that all *E. alata* extracts significantly reduced inflammation by preventing protein denaturation as compared to the reduction in the untreated group (Fig. 1). Acetone demonstrated the highest activity among the extracts, with an IC50 value of 309.45 µg/ml. It was followed by ethanol, which had an IC50 of 426 µg/ml, while the aqueous extract’s IC50 was found to be 504 µg/ml. The abundance and variety of phytochemicals found in *E. alata* could be responsible for the plant’s possible anti-inflammatory capabilities, as demonstrated by its capacity to lessen protein denaturation. Hence, *E. alata* can prove crucial to the treatment of various painful ailments owing to the following problem: while traditional nonsteroidal anti-inflammatory medicines (NSAIDs), including phenylbutazone and indomethazine, function by reducing protein denaturation and suppressing the synthesis of endogenous prostaglandins through the inhibition of the cyclooxygenase enzyme, they may have unfavorable side effects, including ulceration, bleeding, perforation, and blockage^48^.

The World Health Organization predicts that by 2040, 642 million people will be diagnosed with diabetes worldwide^49^. According to a relevant study^50^, high blood sugar levels are the hallmark of diabetes, a metabolic disorder that also affects the kidneys, the nervous system, the heart, and the eyes of humans. Diabetes also increases the chance of leg amputation and blindness. It is estimated that diabetes is going to become more and more common in the future. Notably, approximately 90% of diabetes cases concern type 2 diabetes^49^. In this context, amylase inhibition has been proposed as an important therapeutic target in the management of diabetes and hyperglycemia. Amylase is a digestive enzyme that contributes to the digestion of carbohydrates^32^. To elaborate, the amount of glucose released in the gastrointestinal system is decreased when amylase activity is inhibited^51^.

**Figure 2:**
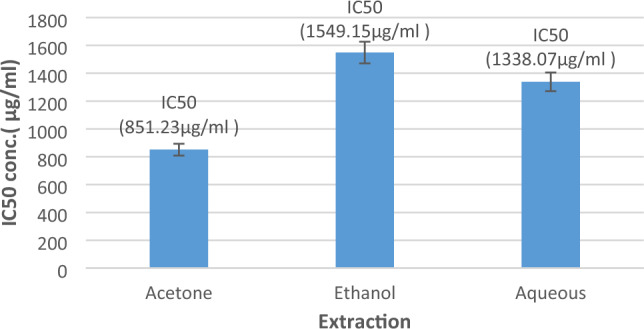
The α-amylase inhibition activity of *E. alata* extracts. The results of α-amylase inhibition are expressed as IC50 (µg/ml); the results are shown as averages for triplicate measurements ± standard deviations (n = 3).

In this light, the results of the current investigation demonstrated that every single *E. alata* extract inhibited amylase (Fig. 2): the ethanol extract showed an IC50 of approximately 1549.15 µg/ml, while the acetone extract had the highest level of inhibition with an IC50 of 851.23 µg/ml. The aqueous extract demonstrated the least efficient inhibition, with an IC50 of around 1338.07 µg/ml. These findings suggest that *E. alata* extracts may reduce the activity of the enzyme amylase, which may prove beneficial in the management of both diabetes and hyperglycemia. According to earlier studies^33,52^, the antihyperglycemic activities of *E. alata* may owe to its metabolite composition. According to another study^31^, the amylase enzyme’s amino acid residues possibly interact with the bioactive compounds in the extracts to decrease their activity and thus interfere with their action. As mentioned in various studies^32,33^, the stoichiometry of interactions between these bioactive compounds and the implicated enzymes may have an impact on the extracts’ ability to block. These results point to a putative mechanism where *E. alata* modulates amylase activity to produce antihyperglycemic benefits.

**Figure 3:**
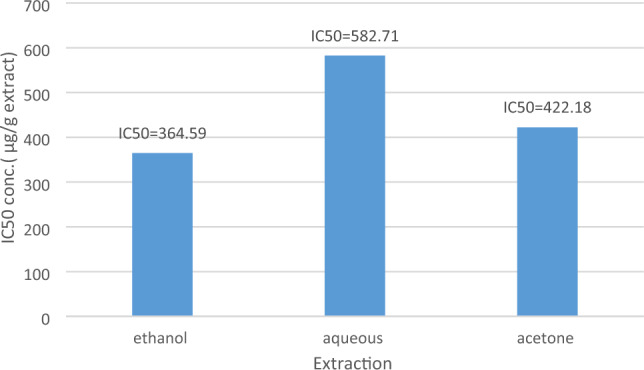
Anti-proliferation activity of *E. alata* extracts. The results of viability inhibition are expressed as IC50 (µg/ml); the results are shown as averages for triplicate measurements ± standard deviations (n = 3).

Moreover, this study focused on the antiproliferative effect of *E. alata* extracts on the MDA-MB-231 breast cancer cell line. The effect of such extracts on the survival of breast cancer cells was assessed using the MTT technique. After 24 h of the cells’ treatment with *E. alata* extracts, the results of the experiment demonstrated a significant dose-dependent inhibition of MDA-MB-231 cell proliferation (Fig. 3). To be precise, for ethanol, acetone, and aqueous extracts, the IC50 values were 364.59 µg/ml, 422.18 µg/ml, and 582.71 µg/ml, respectively. Daunorubicin, the positive control, displayed an IC50 of 1.32 0.05 µg/ml. In addition, MDA-MB-231 cells lost more than 50% of their viability with regard to increases in the treatment doses. At the highest treatment concentration (500 µg/ml), the proportions of MDA-MB-231 cells that multiplied in response to the ethanol and acetone extracts were 38.21% and 43.67%, respectively.

Furthermore, at the maximum treatment concentration (500 µg/ml), the aqueous extract displayed a percentage proliferation of 59.23%. According to these results, *E. alata* extracts may be able to stop breast cancer cells from proliferating. The hydroalcoholic extract of the aerial portion of *E. alata* activates cytotoxic, pro-apoptotic, and anti-proliferative actions on the human breast cancer cell line MCF-7^21^. In contrast to conventional anticancer drugs, however, this extract’s anticancer activity might be only marginally effective. Nevertheless, the current study’s use of *E. alata* extracts revealed the presence of polyphenols, flavonoids, and terpenes therein, in alignment with the findings of past studies. Other relevant studies have demonstrated that terpenes like D-limonene and carvacrol cause apoptosis through the mitochondrial route. Another crucial component, germacrene D, has demonstrated cytotoxic effects on numerous cancer cell lines. ِAdditionally, alpha-tocopherol derivatives reportedly have the ability to kill cells and stop breast cancer cells from surviving, migrating, and invading^53^.

Another study using an ethanolic extract of *A. cherimola*, which is abundant in bioactive compounds (similar to those found in the *E. alata* extracts), reveals that the extract demonstrates specific anti-cancer action against MDA-MB-231 cells. This result confirms the focused effect found in the current study^54^.

## Conclusion

In summary, the current study’s results show the diverse bioactivities of *Ephedra alata Decne* extracts and point to their possible uses as antioxidant, anti-inflammatory, antidiabetic, and anticancer therapeutic products. The study argues that the different types and quantities of phytochemical compounds found in *Ephedra alata Decne* and the observed variations among the bioactivities of various Ephedra extracts may be caused by the solvents used for the purpose of extraction. These compounds reportedly contribute to the therapeutic capabilities of plant extracts and have been recognized in terms of their wide range of biological activities.

The primary biological and phytochemical analyses carried out in this study offer insightful information regarding the possible uses of *Ephedra alata Decne* extracts in the creation of novel medicinal drugs. Both increasing the effectivity of the concomitant medicinal products and improving their extraction process require the possession of knowledge regarding the specific phenolic compounds present in a given extract and their concentrations. Future researchers can delve deeper into these phenolic compounds’ bioactivities and modes of action, uncover their therapeutic potential, and subsequently create new pharmaceuticals or all-natural treatments for a range of illnesses. The results of this study establish the foundation for the further investigation and use of *Ephedra alata Decne* extracts in pharmaceutical industries around the world.

## Data Availability

The datasets used and/or analyzed in the current study can be accessed from the corresponding author upon reasonable request.
